# Cell Cycle Control and DNA Damage Response of Conditionally Immortalized Urothelial Cells

**DOI:** 10.1371/journal.pone.0016595

**Published:** 2011-01-28

**Authors:** Bradley P. Dixon, Jeff Henry, Brian J. Siroky, Albert Chu, Pamela A. Groen, John J. Bissler

**Affiliations:** 1 Division of Nephrology & Hypertension, Cincinnati Children's Hospital Medical Center, Cincinnati, Ohio, United States of America; 2 Division of Pathology & Laboratory Medicine, Cincinnati Children's Hospital Medical Center, Cincinnati, Ohio, United States of America; St. Georges University of London, United Kingdom

## Abstract

**Background:**

Children with complex urogenital anomalies often require bladder reconstruction. Gastrointestinal tissues used in bladder augmentations exhibit a greatly increased risk of malignancy, and the bladder microenvironment may play a role in this carcinogenesis. Investigating the influences of the bladder microenvironment on gastrointestinal and urothelial cell cycle checkpoint activation and DNA damage response has been limited by the lack of an appropriate well-differentiated urothelial cell line system.

**Methodology/Principal Findings:**

To meet this need, we have developed a well-differentiated conditionally immortalized urothelial cell line by isolating it from the *H-2K^b^*-tsA58 transgenic mouse. These cells express a thermosensitive SV40 large T antigen that can be deactivated by adjustment of cell culture conditions, allowing the cell line to regain normal control of the cell cycle. The isolated urothelial cell line demonstrates a polygonal, dome-shaped morphology, expresses cytokeratin 18, and exhibits well-developed tight junctions. Adaptation of the urothelial cell line to hyperosmolal culture conditions induces expression of both cytokeratin 20 and uroplakin II, markers of a superficial urothelial cell or “umbrella cell.” This cell line can be maintained indefinitely in culture under permissive conditions but when cultured under non-permissive conditions, large T antigen expression is reduced substantially, leading to increased p53 activity and reduced cellular proliferation.

**Conclusions/Significance:**

This new model of urothelial cells, along with gastrointestinal cell lines previously derived from the *H-2K^b^*-tsA58 transgenic mouse, will be useful for studying the potential mechanisms of carcinogenesis of the augmented bladder.

## Introduction

Bladder cancer is one of the most common cancers of the urinary tract, with approximately 330,000 new cases worldwide per year [1], and an estimated age-adjusted incidence in the United States of 21.1 per 100,000 men and women per year [2]. Bladder cancer is associated with environmental exposures such as tobacco use [3], occupational carcinogens [4], and infection with *Schistosoma haematobium* [5]. Because many environmental mutagenic compounds are concentrated in the urine, bladder epithelium is frequently exposed to genotoxic stress. The resultant DNA damage must be repaired effectively in order to maintain genomic stability and avoid malignant transformation.

The gastrointestinal tissue portion of bladder augmentations exhibits an eight- to fifteen-fold increased risk of malignancy over that of native bladder tissues [6]. The etiology of this increased risk is poorly understood but may stem from cellular stresses experienced by the non-native bladder tissue in the bladder microenvironment [7]. Both acute exposure and gradual adaptation to hyperosmolal conditions lead to the accumulation of DNA damage and cause this accumulation by disruption of components of the DNA damage response pathway [8], [9]. Urothelial cells lining the mammalian lower urinary tract have adapted to the hyperosmolal urinary microenvironment by organizing into a stratified epithelium, developing tight junctions [10], [11] and forming the asymmetric unit membrane (AUM) consisting of uroplakins [12]. Urothelial cells also accumulate osmolytes such as betaine, myo-inositol, and taurine [13] to balance the effects of hyperosmolality. Using transitional cell carcinoma cell lines, we recently identified that bladder-derived cells maintain the capacity to recognize and repair DNA damage within hyperosmolal microenvironments [14]. Activation of the DNA damage response following adaptation to a hyperosmolal microenvironment appears to be tissue-specific to bladder-derived cells, as we found that these processes are compromised in gastric- and colon-derived adenocarcinoma cell lines [14] under such hyperosmolal conditions. A tissue-specific capacity of urothelial cells to activate the DNA damage response under osmotic stress, and corresponding failure of gastrointestinal cells to do so, may underlie the accumulation of mutations in the gastrointestinal tissues in augmentation cystoplasties [15], potentially leading to their increased risk of carcinogenesis [6], [7].

Unfortunately, the systematic examination of such tissue-specific effects of the bladder microenvironment on DNA damage recognition is hampered by the lack of truly appropriate urothelial models. Cultured primary urothelial cells are highly differentiated [10], but demonstrate phenotypic variability between passages [16], [17], and undergo senescence after a finite number of passages [18], [19]. Urothelial cells immortalized by the wild-type SV40 large T antigen such as the UROtsa and BL-1 cell lines express some markers of urothelium [20], [21], and are phenotypically stable between generations. However, because the large T antigen binds critical proteins such as p53 (for review see Cheng et al [22]), its constitutive expression may interfere with activation of cell cycle checkpoints and apoptosis in response to DNA damage. Similarly, the urothelial cell line derived by Chapman et al is constitutively immortalized by human telomerase reverse transcriptase (hTERT), and although demonstrated to be non-tumorigenic *in vivo* [19], may not be suitable for assessing the cell biology of malignant transformation as telomerase expression alters the expression of genes regulating tumorigenesis [23]. Well-differentiated transitional cell carcinoma cell lines such as RT4 and KK47 [24], [25] also express markers of urothelial differentiation such as uroplakins and cytokeratins [26], [27], but have profound derangements in cell cycle regulation limiting their usefulness as a model of urothelium to study the DNA damage response.

To circumvent the limitations and complement the capacities of current model systems, we developed a conditionally immortalized urothelial cell line derived from *H-2K^b^*-tsA58 mice. Use of these animals to produce primary cell lines that are conditionally immortalized has been well described in the scientific literature [28]–[34]. The derived cell line exhibited differentiation characteristics of urothelium including a polygonal, dome-shaped monolayer of cells with well-developed tight junctions and cytokeratin 18 expression. Cytokeratin 20 and uroplakin II expression could be induced by adaptation of the cells to hyperosmolal culture conditions. In addition, cell cycle control was restored by significant reduction of the SV40 large T antigen and an increase in the cellular activity of p53 under non-permissive conditions as evidenced in part by a decrease in cell proliferation. Likewise the activation of cell cycle checkpoints in response to DNA damage was intact under these latter conditions as was evidenced by S-phase cell cycle arrest following the induction of double strand breaks. To our knowledge, this is the first urothelial cell line that is both well-differentiated and conditionally immortalized, exhibiting normal regulation of cell proliferation and cell cycle checkpoint activation by adjustment of culture conditions. As such, this cell line is an invaluable model of urothelium for studies focused on bladder carcinogenesis, and is the appropriate cell line for comparison to similarly-derived gastrointestinal epithelial cell lines to elucidate the mechanisms underlying the increased risk of malignancy of augmented bladders.

## Methods

### Ethics Statement

The Institutional Animal Care and Use Committee and Institutional Biosafety Committee of the Cincinnati Children's Research Foundation approved all animal experimental procedures (IACUC protocol #9D09068 and IBC Protocol #2009-0086), and these experiments were carried out in accordance with standards as described in the NIH Guide to the Care and Use of Laboratory Animals.

### Preparation of Explant Cultures of Urothelium from Mouse Bladder

The *H-2K^b^*-tsA58 mouse (Immortomouse™), transgenic for the thermosensitive mutant of the SV40 large T antigen (tsA58) expressed by an interferon-inducible MHC Class I promoter, was obtained from Charles River Laboratories, (Wilmington, MA). The tsA58 antigen is ubiquitously expressed, although at the non-permissive body temperature of the mouse (39°C) the protein product of the transgene is unstable and is degraded. Once tissues are removed from the animal, cell lines established from these tissues may be conditionally immortalized under permissive tissue culture conditions of 33°C and in the presence of recombinant mouse interferon gamma (IFN-γ).

ULTI (Urothelial Large T, Inducible) cells were isolated from these mice using a modification of procedures described by Kreft et al [16], [17]. Briefly, following CO_2_ asphyxiation, an incision was made from the symphysis pubis through the sternum, and the urinary bladder was resected in its entirety. Bladders were rinsed with phosphate-buffered saline (PBS), divided sagitally into two equal parts, and mucosa separated from the muscle layer and submucosa. The mucosa was applied to Cyclopore 0.45 µm membrane supports (Becton Dickinson, Franklin Lakes, NJ) in 6 well dishes. Each well contained DMEM:Ham's F12 media (Invitrogen, Carlsbad, CA) supplemented with 10% fetal bovine serum (FBS, Hyclone, Logan, UT), 1% penicillin/streptomycin (Invitrogen), amphotericin B 2.5 µg/ml (Sigma, St. Louis, MO), 1% insulin/transferrin/ethanolamine/selenium (ITES) media supplement (Sigma), mouse epidermal growth factor 10 ng/ml (Sigma), and mouse IFN-γ 10 U/ml (Invitrogen). Cultures were incubated at 5% CO_2_, 37°C for 24 hours, then incubated at 5% CO_2_, 33°C and observed for growth. Media was refreshed from both the inserts and wells twice weekly. Once outgrowth of cells from the mucosal pieces became confluent (at approximately 28 days in culture), the cells were subcultured with 0.25% trypsin and 0.02% EDTA onto 100 mm plastic dishes coated with type I collagen (Becton Dickinson). To drive the ULTI urothelial cells towards a non-transformed phenotype, subcultured cells were grown at 37°C in media identical to that used in the explant experiments, with the exception of the removal of IFN-γ and amphotericin B and the reduction of the FBS concentration to 0.5%.

### Established Cell Lines and Reagents

The RT4 transitional cell carcinoma and NIH 3T3 mouse fibroblast cell lines were obtained from the American Type Culture Collection (ATCC, Manassas, VA) and maintained in cell culture in DMEM:F12 media (Invitrogen) supplemented with 10% FBS and 1% penicillin/streptomycin. Conditionally immortalized gastrointestinal epithelial cell lines ImSt (gastric), YAMC (colon), and MSIE (small intestine), also derived from the *H-2K^b^*-tsA58 mouse, were generous gifts from Dr. Robert Whitehead (Vanderbilt University, Nashville, TN) and were maintained in identical media to our derived urothelial cell line. Etoposide, pifithrin-α and sterile filtered DMSO were obtained from Sigma Chemical.

### Crystal violet cell proliferation assay

ULTI cells were seeded into 96 well plates at a density of 5000 cells/well in complete media containing FBS at concentrations of 10%, 5%, 3%, 2%, 1%, 0.75%, 0.5%, 0.25%, and 0%, both with and without IFN-γ. Cultures containing IFN-γ were incubated at 33°C, and cultures lacking IFN-γ were incubated at 37°C, both for 72 hours. Wells were then washed with PBS, fixed with 4% paraformaldehyde (Electron Microscopy Sciences, Hatfield, PA), washed with ddH_2_O, and incubated with 0.1% crystal violet (Becton Dickinson) for 30 minutes. Cells were again washed with ddH_2_O, and treated with 10% glacial acetic acid. Absorbance was then read at 540 nm with a Bio-Rad Benchmark Plus microplate spectrophotometer. The experiment was carried out in triplicate, and to account for minor variation in initial seeding density between experiments, absorbances from each experiment were normalized to that of 10% FBS in both conditions. Student's t-test was applied between absorbances measured from cells cultured at 33°C and from cells cultured at 37°C for each concentration of FBS to determine statistical significance.

### Cell cycle analysis by flow cytometry

ULTI cells were seeded into 60 mm plastic dishes at a density of 1×10^5^ cells/dish in complete media containing either 10% or 0.5% FBS, both with and without the addition of 10 U/ml IFN-γ. Cultures containing IFN-γ (with either 10% or 0.5% FBS) were incubated at 33°C, and cultures lacking IFN-γ (with either 10% or 0.5% FBS) were incubated at 37°C, each for 72 hours. Cells were then detached with trypsin-EDTA (Invitrogen), pelleted by centrifugation, resuspended in staining buffer consisting of propidium iodide 50 µg/mL, NP-40 0.3%, and RNAse A 1 mg/mL in PBS, and incubated at 4°C for 30 minutes. Cells were then filtered, analyzed with a Becton-Dickinson FACSCanto II cytometer, and the resulting data was interpreted using FlowJo v8.8.6 (TreeStar, Ashland, OR). Experiments were carried out in triplicate, and the mean percentage of cells in G0/G1, S, and G2/M phases were calculated.

In a separate set of experiments, cells were seeded at a density of 1×10^5^ cells/dish in complete media containing either 10% FBS and 10 U/ml IFN-γ, or 0.5% FBS without IFN-γ. Cultures containing IFN-γ and 10% FBS were incubated at 33°C, and cultures without IFN-γ and 0.5% FBS were incubated at 37°C, each for 72 hours. At 60 hours of incubation, cells were treated with etoposide (a potent inhibitor of topoisomerase II that causes double strand DNA breaks) at a final concentration of 25 µM, or a similar volume of the DMSO vehicle. Cells were then detached, pelleted, stained with propidium iodide, and analyzed in the same manner as the previous experiment.

In a third set of experiments, cells were seeded at a density of 1×10^5^ cells/dish in complete media without IFN-γ containing 0.5% FBS and incubated at 37°C for 72 hours. At 54 hours of incubation, cells were treated with either pifithrin-α (a specific inhibitor of p53) at a final concentration of 20 µM or a similar volume of the DMSO vehicle, then at 60 hours of incubation (without washing) treated with etoposide 25 µM or DMSO vehicle. Cells were then detached, pelleted, stained with propidium iodide, and analyzed in the same manner as the previous experiments.

### Western blot analysis

ULTI cells were subcultured onto plastic dishes both in complete media containing 10% FBS as described above, as well as in media lacking IFN-γ and containing 0.5% FBS, at a density of 1.0×10^6^ cells per 100 mm dish. NIH 3T3 fibroblasts were also cultured in standard media as described above. ULTI cultures were either maintained at 33°C in the presence of IFN-γ, or 37°C in the absence of IFN-γ for twenty-four hours, treated with either pifithrin-α 20 µM or DMSO vehicle for six hours, then (without washing) treated with either etoposide 25 µM or DMSO vehicle for twelve hours. Whole cell lysates were then generated by lysis into ice-cold RIPA buffer (50 mM Tris HCl pH 8, 150 mM NaCl, 1% Nonidet-P40, 0.5% sodium deoxycholate, 0.1% SDS) supplemented with protease and phosphatase inhibitors (10 mM NaF, 1 mM Na_3_VO_4_, 1 mM PMSF, 2 µg/ml aprotinin, and 10 µg/ml leupeptin). Cell suspensions were sonicated and cellular debris was pelleted by centrifugation. Supernatants were aliquoted and stored at -80°C.

Protein concentration of these lysates was determined by bicinchoninic acid assay (Pierce, Rockford, IL) according to the manufacturer's instructions. Equal amounts of protein were separated by SDS-PAGE and transferred to Immobilon-P PVDF membranes (Millipore, Billerica, MA). Membranes were blocked with 5% nonfat dry milk in TBS with 0.1% Tween 20 v/v (TBST), then probed with antibody against SV40 large T antigen (1∶2000, Santa Cruz Biotechnology, Santa Cruz, CA); total p53 (1∶40,000), phospho-p53, serine 15 (1∶10,000), cleaved caspase 3 (1∶1000), and PARP (1∶2000) (Cell Signaling Technology, Danvers, MA); p21^cip1^ (1∶1000, BD Pharmingen, San Jose, CA), total ATM (1∶1000, Novus Biologicals, Littleton, CO), phospho-ATM, serine 1981 (1∶1000, Rockland Immunologicals, Gilbertsville, PA), and γH2AX (1∶2000, Trevigen, Gaithersburg, MD). Equal protein loading was confirmed by blotting for G3PDH (1∶40,000; Trevigen). Membranes were then probed with horseradish peroxidase-linked secondary antibody (GE Healthcare Biosciences, Piscataway, NJ), and protein bands detected by chemiluminescence with the Amersham ECL Plus kit (GE Healthcare Biosciences) and developed by autoradiography.

### Phase contrast and scanning electron microscopy of ULTI cells

ULTI cells subcultured onto plastic dishes were examined with Köhler illumination by phase contrast microscopy on a Zeiss Axiovert 200M inverted microscope using the 20x objective. Cells were also subcultured onto Cyclopore 0.45 µm membrane supports (Becton Dickinson), then once the cells reached confluence fixed with 2% (w/v) paraformaldehyde and 2% (v/v) glutaraldehyde in 0.1 M cacodylate buffer, pH 7.4, for 2 hours at 4°C. After rinsing in 0.1 M cacodylate buffer and postfixation with 1% (w/v) OsO_4_, specimens were chemically desiccated with 1,1,1,3,3,3-hexamethyldisilazane (Sigma). Subsequently, the cells were sputter-coated with a gold/palladium 40% mixture in a Denton Vacuum Desk IV, and examined in a Hitachi S-3000N scanning electron microscope.

### Immunofluorescence of cytokeratin 18, ZO-1, and occludin

ULTI cells were seeded onto glass coverslips, and maintained at 37°C in the absence of IFN-γ for four days. Cells were then rinsed with ice-cold PBS, fixed with 4% paraformaldehyde in PBS, and permeabilized with 0.5% Triton X-100 (Sigma) in PBS. Cells were blocked with 15% FBS in PBS, then incubated either with mouse monoclonal anti-cytokeratin 18 antibody (1∶200, Chemicon/Millipore), or a combination of rabbit polyclonal anti-ZO-1 antibody (1∶400, Zymed Laboratories, Carlsbad, CA) and mouse monoclonal anti-occludin antibody (1∶500, Zymed) in PBS with 1% bovine serum albumin (BSA, Sigma) overnight at 4°C. The cells were rinsed with PBS, then incubated with anti-rabbit or anti-mouse secondary antibodies conjugated to Alexa-Fluor 488 and Alexa Fluor 633 (Molecular Probes/Invitrogen) at 1∶500 dilution made in PBS with 1% BSA. Cells were then mounted with ProLong Gold with DAPI (Invitrogen) and coverslips applied to glass slides. Slides were analyzed for cytokeratin 18 with a Zeiss Axiovert 200M inverted fluorescent microscope using 40x and 100x oil-immersion objectives. ZO-1 and occludin expression was assessed with a Zeiss LSM510 confocal microscope equipped with argon 488 nm and HeNe 633 nm laser light sources. Optical sections were taken at 0.5 µm intervals. Orthogonal and z-stack reconstructions were performed with NIH Image J 1.37a software.

### Reverse transcriptase PCR of cytokeratin 18, cytokeratin 20, and uroplakin II

ULTI cells as well as YAMC, MSIE, and ImSt gastrointestinal cells and NIH 3T3 fibroblasts were seeded into type I collagen-coated plastic dishes with media as described above without IFN-γ, and maintained at 37°C for four days. Cells were scraped into ice-cold sterile PBS, pelleted, and total cellular RNA was extracted from pelleted cells using TRI reagent (Sigma) according to the manufacturer's instructions and quantitated by spectrophotometry.

In experiments designed to mimic the hyperosmolal bladder microenvironment, ULTI cells were gradually adapted to hyperosmolality in culture by increasing the media osmolality by 50 mOsm/kg every 24 hours from a basal media osmolality (∼300 mOsm/kg) to target osmolalities of 450 and 600 mOsm/kg. Cells were seeded into plastic dishes with media lacking IFN-γ and containing 0.5% FBS and maintained at 37°C. Every 24 hours, culture media was aspirated and replaced with fresh media adjusted to the corresponding osmolality by the addition of sterile-filtered 5M NaCl (Sigma) or 5M urea (Fluka). To remove isocyanates, a degradation product of urea, 20 mL of 5M urea stock solution was exchanged with 1 g of AG-501-X8 resin (Bio-Rad) in the method published by Zhang et al [35] immediately prior to use. Cells were scraped into PBS and total cellular RNA extracted with TRI reagent as above.

To provide tissue RNA for comparison, total cellular RNA was extracted with TRI reagent from homogenized stomach, intestine, heart, and bladder tissue from euthanized wild-type mice.

Following treatment of 0.3–1 µg of extracted RNA with 2 Units of RNAse-free DNAse I (New England Biolabs, Ipswich, MA) and heat deactivation of the DNAse, cDNA was generated by reverse transcription using oligo-dT_20_ primers and the SuperScript III First-Strand Synthesis System (Invitrogen) according to the manufacturer's instructions. Aliquots of cDNA were then used to detect cytokeratin 18, cytokeratin 20, uroplakin II, and G3PDH by PCR with AccuPower PCR PreMix tubes (Bioneer, Alameda, CA). Forward and reverse primer sets, PCR reaction annealing temperature, and predicted product sizes are listed in Table 1. The PCR program template for all reactions was as follows: 94°C×5 minutes, followed by forty cycles of (94°C×1 minute, annealing temperature×30 seconds, 72°C×30 sec), and final extension of 72°C×10 minutes. PCR products were separated by agarose gel electrophoresis, stained with SYBR® Safe (Invitrogen) and imaged.

**Table 1:** Reverse transcriptase PCR primer pairs, annealing temperature (T_annealing_), and predicted PCR product size for cytokeratin 18, cytokeratin 20, uroplakin II, and glyceraldehyde-3-phosphate dehydrogenase (G3PDH).

Primer Pairs	T_Annealing_	Predicted Product Size
*Mus musculus* Cytokeratin 18 (*Krt18*)	58°C	469 bp
Forward TTTAGAGTCAAGTATGAGAC		
Reverse AGTTGATGTTCTGGTTTTTC		
*Mus musculus* Cytokeratin 20 (*Krt20*)	52°C	467 bp
Forward TCAGATTGAAGTTTGAGACT		
Reverse CAGAGACTCTTTCATGCTGA		
*Mus musculus* Uroplakin II (*Upk2*)	65°C	233 bp
Forward CGACAGCAAAGTGGTTAAGT		
Reverse CCATGTTTTTTCGAGGAAGC		
*Mus musculus* G3PDH (*Gapdh*)	58°C	752 bp
Forward AGGTCGGTGTGAACGGATT		
Reverse ATACTTGGCAGGTTTCTCCA		

## Results

### ULTI cells regulate cell proliferation normally under non-permissive conditions

**Figure 1:**
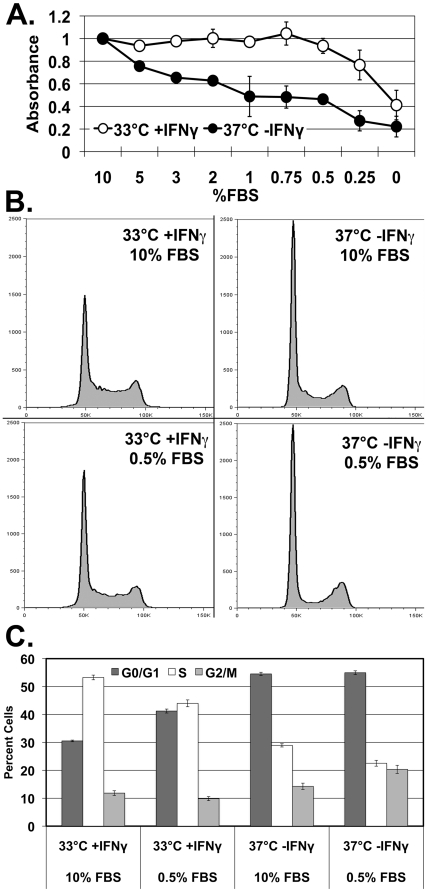
The ULTI mouse urothelial cell line is conditionally immortalized under permissive conditions, but restores cell cycle control under non-permissive conditions. A) Crystal violet proliferation assay, in which absorbances were normalized to that of 10% FBS to reduce interexperimental variability. Open circles represent cells grown under permissive conditions (33°C +IFN-γ), whereas closed circles represent cells grown under non-permissive conditions (37°C -IFN-γ). Error bars represent standard error of the mean. B) Cell cycle analysis of ULTI cells under both permissive and non-permissive conditions, and with 10% and 0.5% FBS concentration, by propidium iodide DNA labeling flow cytometry. C) Quantitation of cell cycle phase of ULTI cells under both permissive and non-permissive conditions, and with 10% and 0.5% FBS concentration. Dark grey bars indicate percent of cells in G0/G1, white bars indicate percent of cells in S phase, and light grey bars indicate percent of cells in G2/M phase. Error bars represent standard error of the mean.

Cells grown in permissive conditions for SV40 large T antigen expression (33°C +IFN-γ) continued to show robust proliferation as measured by crystal violet assay despite a reduction in FBS concentration from 10% through to 0.5% (Figure 1a). However, cells grown in non-permissive conditions (37°C -IFN-γ) had a normal stepwise reduction in cell proliferation at each decreasing concentration of FBS. The difference in absorbance between cells cultured in permissive versus non-permissive conditions was statistically significant (p<0.05) at 3%, 2%, 0.75%, and 0.5% FBS.

Aneuploidy was excluded in cells grown under both permissive and non-permissive conditions by cell cycle analysis with flow cytometry using propidium iodide DNA labeling (Figure 1b). Cells cultured under non-permissive conditions with either 10% or 0.5% FBS demonstrated a reduction in the proportion of cells in S-phase and an increase of cells in G0/G1 compared to cells cultured at permissive conditions with the same FBS concentration (Figure 1c), indicating proliferation may be manipulated by inducing or suppressing SV40 large T antigen expression.

### The DNA damage response of ULTI cells is unaffected by conditional immortalization

**Figure 2:**
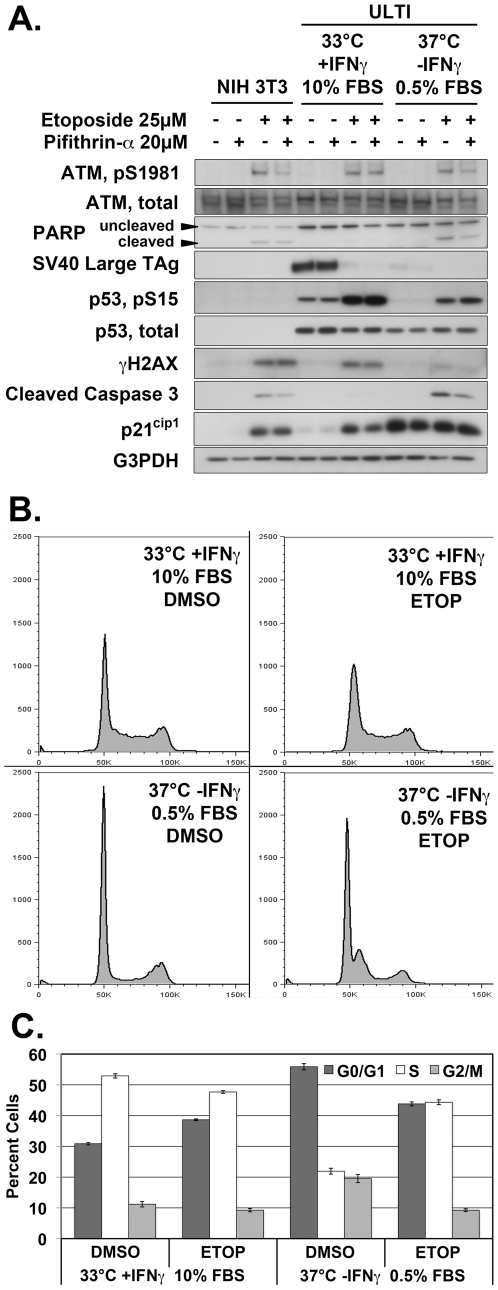
The ULTI mouse urothelial cell line has an intact DNA damage response under permissive conditions, but aberrant cell cycle checkpoint and apoptosis activation which normalizes under non-permissive conditions. A) Western blot of whole cell lysates from NIH 3T3 fibroblasts or ULTI cells under both permissive and non-permissive conditions, pretreated with pifithrin-α or DMSO vehicle, then exposed to etoposide or DMSO vehicle. B) Cell cycle analysis by propidium iodide DNA labeling flow cytometry of ULTI cells treated with etoposide (ETOP) or DMSO vehicle under both permissive conditions with 10% FBS, and non-permissive conditions with 0.5% FBS. C) Quantitation of cell cycle phase of ULTI cells treated with etoposide (ETOP) or DMSO vehicle under both permissive conditions with 10% FBS and non-permissive conditions with 0.5% FBS. Dark grey bars indicate percent of cells in G0/G1, white bars indicate percent of cells in S phase, and light grey bars indicate percent of cells in G2/M phase. Error bars represent standard error of the mean.

The DNA damage response pathway was assessed by western blot analysis in whole cell lysates from ULTI cells exposed to etoposide under both permissive and non-permissive conditions, as well as NIH 3T3 cells. This pathway was appropriately activated following induction of DNA damage with etoposide in ULTI cells cultured under both permissive and non-permissive conditions as determined by both autophosphorylation of the ataxia telangectasia mutated (ATM) kinase on serine 1981 and phosphorylation of H2AX on serine 319 (γH2AX) by ATM (Figure 2a). This activation was increased under permissive conditions when compared to non-permissive conditions, which could be explained by progression through the cell cycle despite the presence of double strand DNA breaks.

SV40 large T antigen expression was substantially reduced in cells grown under non-permissive conditions compared to cells in permissive conditions (Figure 2a), confirming the conditional transformation of this cell line. Treatment of cells with etoposide also caused a substantial reduction in cellular levels of SV40 large T antigen. The large T antigen is known to undergo targeted proteasomal degradation as part of the DNA damage response [36].

### Cell cycle checkpoint activation and apoptosis are inhibited under permissive conditions through a functional inactivation of p53

Levels of p53 were increased in cells grown under permissive conditions compared to cells grown under non-permissive conditions (Figure 2a), indicating sequestration and decreased degradation of p53 due to its binding to SV40 large T antigen at permissive conditions. The phosphorylation of p53 on serine 15 increased with etoposide exposure under both permissive and non-permissive conditions (Figure 2a). However, levels of p21, an effector of cell cycle arrest whose expression is induced by p53, were significantly increased in cells grown under non-permissive conditions when compared to those grown under permissive conditions, although p21 expression increased following exposure to etoposide under permissive conditions (Figure 2a).

As measured by flow cytometry, G1/S-phase cell cycle checkpoint activation was attenuated under permissive conditions (Figure 2b, upper panels). Cells exposed to DNA damage induced by etoposide under these conditions had similar proportions of cells in S-phase comparing etoposide treatment (52.9%) to control (47.7%, Figure 2c). In contrast, cells cultured under conditions non-permissive for large T antigen expression and exposed to etoposide displayed a prominent S-phase arrest (Figure 2b, lower panels), with a significant increase in the population of S-phase cells in response to damage (from 21.9% to 44.3%) indicating activation of this cell cycle checkpoint (Figure 2c).

**Figure 3:**
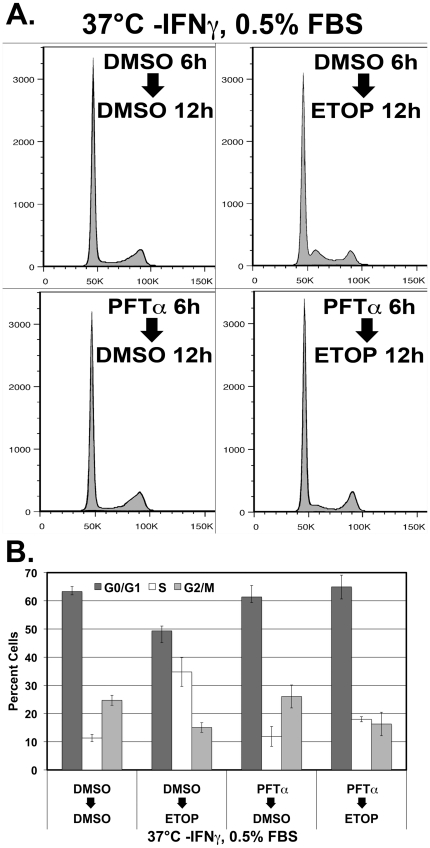
The G1/S cell cycle checkpoint activation of the ULTI mouse urothelial cell line under non-permissive conditions is sensitive to p53 inhibition. A) Cell cycle analysis by propidium iodide DNA labeling flow cytometry of ULTI cells pretreated with pifithrin-α (PFTa) or DMSO vehicle, then treated with etoposide (ETOP) or DMSO vehicle under non-permissive conditions with 0.5% FBS. B) Quantitation of cell cycle phase of ULTI cells pretreated with pifithrin-α (PFTa) or DMSO vehicle, then treated with etoposide (ETOP) or DMSO vehicle under non-permissive conditions with 0.5% FBS. Dark grey bars indicate percent of cells in G0/G1, white bars indicate percent of cells in S phase, and light grey bars indicate percent of cells in G2/M phase. Error bars represent standard error of the mean.

The checkpoint activation under non-permissive conditions was sensitive to p53 inhibition. Pretreatment with pifithrin-α led to a diminution of the S-phase arrest (Figure 3a, lower panels) and reduction in the S-phase population of cells damaged with etoposide (18.0%, Figure 3b) when compared to cells pretreated with DMSO vehicle then damaged with etoposide (34.8%). The reduction in S-phase arrest in cells cultured under non-permissive conditions, pretreated with pifithrin-αthen damaged with etoposide indicates that p53 is functionally active under non-permissive conditions and mediates the S-phase arrest.

Irreversible DNA damage that accumulates to a critical threshold triggers apoptosis, mediated in large part by p53 and induction of its effectors, PUMA and Bax. Cells damaged with etoposide under permissive growth conditions had a substantial decrease in activation of the apoptotic pathway as determined by cleavage of caspase 3, a key step at the convergence of the intrinsic and extrinsic apoptosis pathways, and poly-ADP ribose polymerase (PARP), a downstream substrate of caspase 3, when compared to cells damaged under non-permissive conditions (Figure 2a). The activation of the apoptotic pathway in cells damaged with etoposide under non-permissive conditions was blunted by pretreatment with pifithrin-α, indicating p53 is indeed a mediator of DNA damage-induced apoptosis under such conditions.

NIH 3T3 fibroblasts, used as a control murine cell line with wild-type p53 and lacking SV40 Large T antigen expression, demonstrated a normal activation of ATM, γH2AX, increase in p21 abundance, cleavage of caspase 3, and PARP following exposure to etoposide (Figure 2a). Cellular abundance of p53 was much less in the NIH 3T3 cells than in the ULTI cell line, but both total p53 levels and phosphorylation of p53 on serine 15 were found to be appropriately increased following damage with etoposide (data not shown).

### ULTI cells have an epithelial morphology consistent with bladder urothelium, and express epithelial protein markers

**Figure 4:**
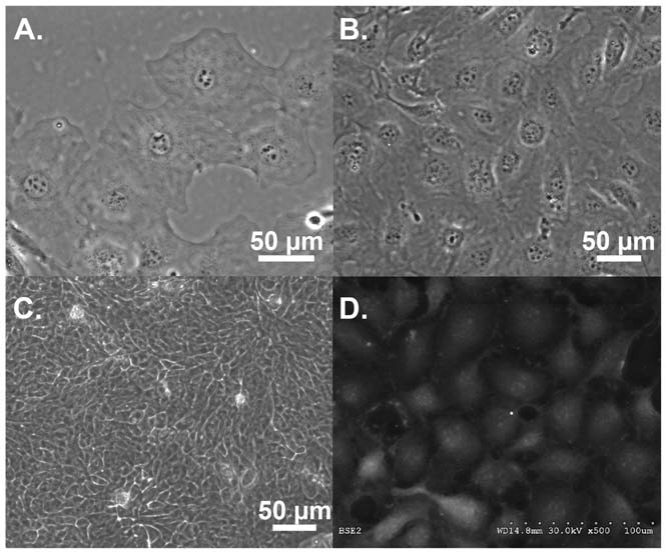
The ULTI mouse urothelial cell line exhibits an epithelial morphology. A) Phase contrast micrograph obtained with 20X objective of a monolayer of ULTI cells, showing a polygonal morphology. B) Phase contrast micrograph obtained with 20X objective of ULTI cells demonstrating the organization of these cells into densely packed sheets. C) Phase contrast micrograph obtained with 20X objective of a confluent monolayer of RT4 transitional cell carcinoma cell line, also demonstrating a densely packed arrangement of smaller cells. D) Scanning electron micrograph (500X) of ULTI cells, showing a domed, polygonal morphology with smooth apical surface. Bar at the bottom right of the panel represents 100 µm.

Phase contrast images of the ULTI cell line revealed a monolayer of domed, polygonal cells with epithelial morphology (Figure 4a). These cells were arranged in tight clusters (Figure 4b), suggestive of mature tight junctions and focal adhesions [37]. This cluster arrangement is characteristic of other cell lines of urothelial origin such as the transitional cell carcinoma line RT4 (Figure 4c), which is by comparison smaller than primary urothelial cells [24]. Scanning electron microscopy of ULTI cells also demonstrated a densely packed monolayer of domed polygonal cells (Figure 4d), although the cell surface appeared smooth, lacking ridges consistent with the AUM in fully differentiated urothelial or “umbrella” cells.

**Figure 5:**
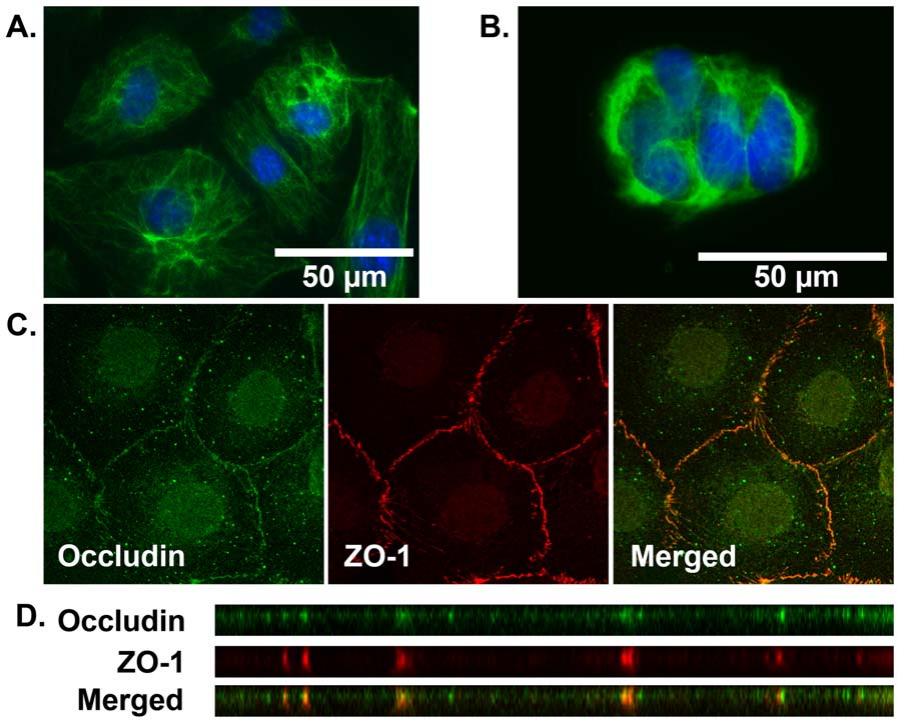
The ULTI cell line expresses epithelial markers of differentiation. A) Cytokeratin 18 immunofluorescence of ULTI cells, obtained with 40X objective. Filamentous staining is noted characteristic of the cytokeratins. B) Cytokeratin 18 immunofluorescence of RT4 transitional cell carcinoma cells, obtained with 40X objective. Similar staining to ULTI cells is noted. Withholding primary antibody to assess nonspecific binding of the secondary antibody found no such staining. C) Confocal immunofluorescence of ULTI cells. Circumferential linear staining is noted with occludin (green, left panel) and ZO-1 (red, middle panel), which colocalizes upon merge of the images (yellow, right panel). D) Orthogonal reconstruction of axial images demonstrating colocalization of occludin and ZO-1.

ULTI mouse urothelial cells expressed cytokeratin 18 in a cytoplasmic distribution characteristic of the intermediate filament compartment (Figure 5a). A similar staining pattern was noted in the RT4 transitional carcinoma cell line (Figure 5b), supporting the epithelial nature of the derived mouse cell line. To further define the epithelial nature of these cells, expression of the tight junction proteins ZO-1 and occludin were characterized by confocal immunofluorescent microscopy. The ULTI cell line exhibited linear staining for both ZO-1 and occludin in a circumferential pattern (Figure 5c), which colocalized both in the axial plane and upon orthogonal reconstruction of the confocal images (Figure 5d). RT4 cells also demonstrated a similar circumferential staining with ZO-1 and occludin, whereas the 3T3 fibroblast cell line demonstrated discrete staining only at focal cell-cell junctions (data not shown).

### Differentiation state of ULTI cell line

**Figure 6:**
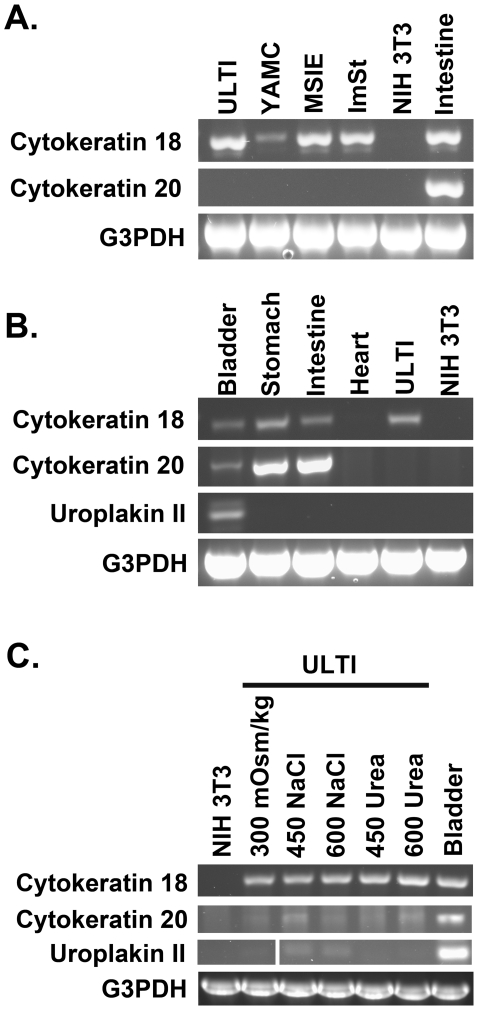
ULTI cells express cytokeratin 20 and uroplakin II in addition to cytokeratin 18 under hyperosmolal conditions. A) RT-PCR of cytokeratin 18, cytokeratin 20, and G3PDH using RNA isolated from cell lines. B) RT-PCR of cytokeratin 18, cytokeratin 20, uroplakin II, and G3PDH from RNA isolated from mouse tissues and ULTI cell line. C) RT-PCR of cytokeratin 18, cytokeratin 20, uroplakin II, and G3PDH from RNA isolated from ULTI cell line under basal and hyperosmolal conditions and NIH 3T3 cells. The panel displaying the uroplakin II PCR products is composed of two images from two separate areas of the same gel image.

To further define the differentiation state of the ULTI cell line, gene expression analysis for cytokeratins and uroplakin was undertaken using RT-PCR. These studies compared expression from the ULTI cell line as well as other conditionally immortalized epithelial cell lines (YAMC, MSIE, ImSt) and from various mouse tissues. Both the ULTI cell line and the conditionally immortalized gastrointestinal cell lines expressed cytokeratin 18, but did not express cytokeratin 20 (Figure 6a) under standard non-immortalized culture conditions. Mouse stomach, intestine and bladder tissue all demonstrated expression of both CK18 and CK20 (Figure 6b). Uroplakin II expression was limited to mouse bladder tissue and was not expressed by the ULTI cell line under standard conditions (Figure 6b). Following gradual adaptation of the ULTI cells to 450mOsm/kg and 600mOsm/kg with sodium chloride, but not urea, expression of uroplakin II was detected (Figure 6c). Cytokeratin 20 expression was also following adaptation to 450mOsm/kg and 600mOsm/kg with sodium chloride as well as urea (Figure 6c). Amplification of G3PDH confirmed equal loading of RNA and generation of cDNA (Figures 6a, 6b, and 6c).

## Discussion

In order to develop meaningful strategies aimed at the prevention and treatment of bladder cancer, and especially cancer of the augmented bladder, a greater understanding of the role of the DNA damage response, DNA repair, activation of cell cycle checkpoints and apoptosis, and the influence of the bladder microenvironment on these processes in both native bladder and gastrointestinal tissues is critical. Population based genetic studies have indicated a role for DNA repair proteins like ERCC1 [38] as well as the *MYC*, *TP63*, and *PSCA* loci (reviewed in [39]) in the pathogenesis of bladder cancer but unfortunately, there are no suitable *in vitro* models of urothelium that have proper regulatory control of cell cycle checkpoints and apoptosis as well as an intact DNA damage response.

To address this limitation, we have developed a conditionally immortalized mouse urothelial cell line that exhibits an epithelial morphology (Figure 4), formation of tight junctions (Figure 5), and expression of urothelial markers such as cytokeratin 18. (Figures 5 and 6). Under the standard isoosmolal culture conditions used for the cell proliferation, DNA damage response, and cell cycle analysis experiments, ULTI cells did not terminally differentiate as they lacked expression of cytokeratin 20 [40] or uroplakin II [11] (Figure 6a and 6b). A similar pattern of intermediate differentiation under standard culture conditions has been observed with other conditionally immortalized cell lines such as the gastrointestinal YAMC, MSIE, and ImSt cell lines [41] and even successive passages of primary urothelial cells [17], [42]. As expression of both cytokeratin 20 and uroplakin II were detected following adaptation to a hyperosmolal milieu (Figure 6c), chronic osmotic stress may serve as a differentiation stimulus in these cells towards an “umbrella cell” phenotype, suggesting that induction of cytokeratin 20 and uroplakin expression in this cell line may be linked to activation of elements of the osmotic stress response by proteins such as TonEBP [43].

This urothelial cell line can be subcultured more than 50 passages under permissive cell culture, but has a stable phenotype under non-permissive conditions. The conditional transformation of ULTI cells was confirmed as cells under permissive conditions displayed robust proliferation over a wide range of FBS concentrations (Figures 1a and 1b), indicating that the induced expression of SV40 large T antigen is the primary determinant of cell proliferation under these conditions. Cells grown under non-permissive conditions showed an expected stepwise decrease in cell proliferation with decreasing FBS concentration, indicating normal cell cycle control and regulation of proliferation in response to the reduction of growth factors.

ULTI cells demonstrated an appropriate activation of the DNA damage response pathway mediated by ATM and its downstream targets such as γH2AX in cells exposed to etoposide under both permissive and non-permissive conditions. However, only ULTI cells exposed to etoposide under non-permissive conditions activated a G1/S-phase cell cycle checkpoint, and activated mediators of apoptosis such as caspase-3 and PARP (Figures 2a and 2b), whereas cells under permissive conditions failed to respond in this normal fashion. Levels of p53 were higher under permissive conditions corresponding to increased expression and stability of the SV40 large T antigen and subsequent binding to, and sequestration of, p53 [22]. However, levels of p21 were dramatically increased in the cells under non-permissive conditions despite decreased abundance of p53. This finding is expected because intracellular levels of p21 are affected by other regulators of the cell cycle, such as p16*^INK4a^* [44], and are involved in cellular quiescence [45]. ULTI cells under non-permissive conditions with very low (0.5%) FBS concentration had decreased proliferation (Figure 1a) and an increased population of G0/G1 cells (Figure 1b), supporting the likelihood that p21 expression was induced in such a p53-independent manner.

Phosphorylation of p53 on serine 15 following etoposide treatment occurred under both permissive and non-permissive conditions (Figure 2a). Although the overall abundance of p53 was greater under permissive conditions, the degree of change between DMSO and etoposide treatment was much greater under non-permissive conditions indicating a more sensitive detection of DNA damage under non-permissive conditions mediated by p53. Selective inhibition of p53 activity with pifithrin led to a decreased level of cleaved caspase 3 and cleaved PARP under non-permissive conditions following etoposide treatment (Figure 2a), as well as a blunted activation of the G1/S cell cycle checkpoint (Figures 3a and 3b). This confirms that, under such conditions, p53 plays a critical role in the activation of cell cycle checkpoints and apoptosis due to DNA damage induced by etoposide. This finding supports the notion that p53 is no longer sequestered by the SV40 large T antigen and would therefore be able to induce expression of its apoptotic effectors such as Bax and PUMA.

In conclusion, the ULTI mouse urothelial cell line is both well-differentiated and conditionally immortalized due to the ability to modulate cellular levels of the SV40 large T antigen following adjustment of the cell culture milieu. Although the DNA damage response mediated by ATM is intact in these cells under both permissive and non-permissive conditions, activation of cell cycle checkpoints and apoptosis may be inhibited by the induced expression of the SV40 large T antigen under permissive conditions promoting cell proliferation despite the presence of DNA damage. These pathways are restored when cells are grown under non-permissive conditions. This reversible immortalization is unique to this urothelial cell line, and makes this cell line the optimal model for the characterization of cell cycle checkpoint activation, apoptosis, and the DNA damage response in urothelium. This cell line will be critical in experiments aimed at providing insight into the effect of bladder microenvironment on the DNA damage response and into the pathomechanisms leading to carcinogenesis of native and augmented bladders.
